# The role of honour in interpersonal, intrapersonal and intergroup processes

**DOI:** 10.1111/spc3.12719

**Published:** 2022-12-11

**Authors:** Ayse K. Uskul, Susan E. Cross, Ceren Günsoy

**Affiliations:** ^1^ School of Psychology University of Sussex Falmer, Brighton UK; ^2^ Department of Psychology Iowa State University Ames Iowa USA; ^3^ Department of Psychology University of Rhode Island Kingston Rhode Island USA

**Keywords:** culture, honor, interpersonal, intrapersonal, intergroup processes

## Abstract

In this article, we review research in psychology and other related social science fields that has adopted an honor framework to examine intrapersonal, interpersonal, and intergroup processes taking a culture‐comparative or individual differences approach. In the sections below, we will first review research on the role of honor in interpersonal processes focusing primarily on interpersonal aggression including in close relationships, non‐aggressive ways of responding to threats (e.g., forgiveness), and reciprocity. Next, we move onto reviewing research on the role of honor in intrapersonal processes, specifically in the domains of emotional responses to honor‐threatening situations, mental, and physical health. Finally, we review research emerging from social and political psychology and political science that have utilized the honor framework to understand and explain group processes and intergroup relations at different level of analyses (e.g., social groups, nations). Given the limited space, our goal was to emphasize major and emerging areas of research on honor and provide food for thought for future research.

## 1 THE ROLE OF HONOR IN INTERPERSONAL, INTRAPERSONAL AND INTERGROUP PROCESSES

In this article, we review research in psychology and other related social science fields that has adopted an honor framework to examine interpersonal (occurring between individuals), intrapersonal (occurring within an individual's own mind), and intergroup (occurring between members of groups) processes taking a culture‐comparative or individual differences approach. In the sections below, we first review research on the role of honor in interpersonal processes focusing primarily on interpersonal aggression including in close relationships, non‐aggressive ways of responding to threats, and reciprocity. Next, we focus on the role of honor in intrapersonal processes, specifically in the domains of emotional responses to honor‐threatening situations, mental, and physical health. Finally, we review research emerging from psychology and political science that has utilized an honor framework to understand and explain group processes and intergroup relations at different level of analyses (e.g., social groups, nations). Given the limited space, our goal was to emphasize major and emerging areas of research on honor and provide food for thought for future research, and we recommend readers consult other review pieces (e.g., Cross & Uskul, 2022; Gul et al., 2021; Uskul & Cross, 2019; Uskul et al., 2019) for a more comprehensive list of readings in this area. We finish by highlighting that honor's role in psychological processes can take various forms depending on individuals' cultural background, features of the situation, and individual differences in honor endorsement.

### 1.1 What is honor?

The concept of honor was introduced into the social science literature in the 1960's with an influential volume edited by John Peristiany (1965) titled *Honor and Shame, The Values of the Mediterranean Society*. Contributors to the volume described honor as a value that is “…at the apex of the social pyramid of temporal social values” (Peristiany, 1965, a:10) and as comprising both an individual's sense of self‐worth and their reputation in the surrounding community (Pitt‐Rivers, 1965). Honor was also discussed as a highly‐gendered value, applying to both men and women, albeit under different sets of socially approved rules, and as a relational construct situated within the context of family, kinship, local communities and larger social units. Since the publication of this volume, honor has generally been conceptualized as self‐worth in one's own eyes and in the eyes of others (Pitt‐Rivers, 1965), thus to be honorable was taken as to have self‐esteem or self‐respect (e.g., to be proud of one's personal accomplishments) and to be known by others as a respectable and moral person (Cross et al., 2014; Cross & Uskul, 2022). Social scientists have employed honor in their research either as an individual difference variable, especially in work that is conducted within a single cultural context, or as a variable that is more or less characteristic of a given cultural context when compared with other cultural contexts, as has typically been done in comparative designs, as we discuss in greater detail below.

### 1.2 The cultural logic of honor

More recently, in social psychology honor has been conceptualized as a type of cultural logic (along with cultural logics of face and dignity, see Leung & Cohen, 2011 for definitions of these constructs and how they are similar to or different from the cultural logic of honor) which refers to a cognitive structure that helps beliefs, values and practices in a given cultural context fit together into a coherent whole which is then used to make sense of the social world and respond appropriately to situations (Leung & Cohen, 2011). Societies that promote a cultural logic of honor (e.g., Latin American, Middle Eastern, North African, South Asian regions) are thought to emerge in locations where resources are portable and the rule of law is unavailable or weak (Edgerton, 1971; McWhiney, 1988). In such societies, individuals develop social psychological tools that help them gain a reputation for toughness and strength and a willingness to retaliate to threats directed to their livelihood and reputation. These contexts then breed a cultural logic in which one's view of oneself (individual worth), one's social reputation (social worth and status), and the societal expectations against which one's behavior is assessed work together in shaping an individual's honor (Cross et al., 2014; Peristiany, 1965; Pitt‐Rivers, 1965). Honor in these societies is experienced as socially fragile; it can be lost through an individual's actions, and once lost, can be difficult to regain. As a result, individuals in cultural groups that adopt a cultural logic of honor feel motivated to maintain and protect their honor by reciprocating positive and negative actions and by being vigilant for attacks directed to their honor and responding to honor threats in ways (e.g., by retaliating) that make others reluctant to antagonize them in the future. Honor is also highly relational in that an individual's own (negative and positive) behavior is likely to shape the honor of close others or ingroup members and, conversely, close others and ingroup members' behaviors are likely to spill over to threaten or enhance an individuals' honor (e.g., Rodriguez Mosquera., 2014; Uskul et al., 2012). Finally, honor has been considered to be a multifaceted construct involving subcomponents such as integrity, family honor and more gendered forms such as masculine and feminine honor that have been operationalized and assessed as values, concerns, ideologies, and beliefs.

Social science research has examined honor as a value, concern, and societal norm in relation to a wide range of outcome variables taking a culture‐comparative and individual difference approach, using the construct to both distinguish between cultural groups (e.g., Smith et al., 2017; Yao et al., 2017) and discriminate between individuals who endorse honor to different degrees (e.g., Frey et al., 2021). Research on honor started out to examine interpersonal outcomes, mainly aggression in the face of honor threats, but has more recently moved onto becoming more varied in its focus to also include intrapersonal and intergroup processes. This review provides a brief account of this research highlighting the theoretical and explanatory value of considering honor as a construct in the examination of a wide range of processes at different levels of analyses.

## 2 HONOR IN INTERPERSONAL PROCESSES

In societies shaped by concerns for honor and social image, interpersonal relations will be marked by attention to the implications of the situation for one's reputation. These societies expect individuals to respond quickly and assertively to threats to their reputation, to demonstrate that they are not someone who can be disrespected, and to show that they are willing to retaliate against such threats. Research in the last three decades has revealed that these expectations influence interpersonal behavior in a variety of areas, some of which we cover below.

### 2.1 Honor and interpersonal aggression

Much of the initial research by psychologists on cultures of honor focused on interpersonal responses to threats to one's reputation. Cohen et al.’s (1996) ground‐breaking experimental ethnography showed that when insulted, men from honor states in the southern U.S. responded more aggressively than did men from dignity states in the northern U.S. Analyses of archival data showed that White men in southern states were more likely to respond to an affront with violence compared to White men in northern states, as indicated by homicide rates (Nisbett & Cohen, 1996) and rates of school shootings by adolescent boys (Brown et al., 2009). This relation between honor threat and aggressive behavior has also been identified among Turkish participants (Uskul et al., 2015) and Dutch‐Turkish participants (van Osch et al., 2013).

The link between honor threat and aggression is embedded in people's attitudes and perceptions of social norms. For example, U.S. Southerners endorse violence to protect one's family or reputation more than do U.S. Northerners but there is no north‐south difference in endorsement of other kinds of violence (Cohen & Nisbett, 1994). U.S. students who strongly endorse masculine honor beliefs (O’Dea et al., 2017, 2018), U.S. southerners (Cohen et al., 1996), and Turkish college students (Cross et al., 2013) approve of men who confront an attacker or who fight back more than do their non‐honor‐oriented comparison groups. Men are often the ones expected to promote and protect their own and their family's honor through aggressive retaliation, but honor‐oriented women may also endorse forms of aggression consistent with gender roles. Indeed, U.S. southern women who endorsed feminine honor norms were more likely to endorse forms of reactive relational aggression (such as ostracism) in response to situations that offended or insulted the individual (Foster et al., 2022; see also Chalman et al., 2021).

#### 2.1.1 The case of close relationships

The interdependent nature of honor means that behaviors of those who are close to us can have implications for us. In addition, honor cultures often have divergent expectations for men and women, which include men's authority over women and women's loyalty and sacrifice for their family. Consequently, gendered honor norms can allow men in close relationships to justify aggression against women when they perceive that the woman has threatened their partner's, father's, or family's honor and reputation (e.g., through disobedience or infidelity; Vandello & Cohen, 2003, 2008; Vandello et al., 2009).

For example, Vandello and colleagues (Vandello & Cohen, 2003; Vandello et al., 2009) found that members of honor cultures (Brazilians, Chileans, U.S. Southerners, Latinx Americans) compared to members of dignity cultures (U.S. northerners), perceived a wife's infidelity as more damaging to her husband's honor and were less critical of the husband's aggressive response to the infidelity. In another study, when asked to evaluate a situation in which a man rapes his wife after learning of her infidelity, Turkish participants (an honor culture) were more likely to blame the victim and less likely to blame the perpetrator or to label the incident as rape, compared to participants from the U.K. or Germany (Gul & Schuster, 2020). Similarly, among men in India who were asked to evaluate how they would respond to a daughter who transgressed gender norms (e.g., by getting drunk or having premarital sex), men who strongly endorsed the goal of restoring their honor tended to report that they would slap or disown their daughter, especially if others in the community were aware of the transgression (Ashokkumar & Swann, 2022). In short, considerable research shows that in honor‐focused contexts, violence towards close others may be endorsed to restore honor and social standing following their dishonorable behavior.

### 2.2 Other responses to interpersonal threats to honor

Aggression is one of several possible reactions available to people who strongly endorse honor values (or who live in contexts where those values are emphasized) after a threat to their honor. An individual could also choose not to respond aggressively, but instead forgive the offender. Forgiveness is widely viewed by Western theorists and researchers as a positive behavior that benefits the forgiver (Bono et al., 2008; Karremans et al., 2003); however, researchers have seldom investigated forgiveness in honor contexts where individuals may perceive that forgiving an offender will mark them as weak or without honor (Ceylan‐Batur et al., 2022; see also Williamson et al., 2014). Studies focused on cultures of honor have shown that Latinx Americans were less likely to forgive offenses that had harmed their reputation than were European Americans (Castillo, 2019) and that Mexican participants were less likely than participants from northern U.S. to forgive an offense that had harmed their reputation if the offender did not apologize or engage in other reparative behavior (Castillo, 2022). In short, in honor contexts, forgiveness may be in short supply for offenses that threaten the victim's reputation or when the apology makes the offender appear weak or dishonorable.

### 2.3 Honor and reciprocity

As shown in studies on responses to honor threats, reciprocity or “payback” is a key feature of cultures of honor, yet payback has two faces in cultures of honor: Individuals are expected to retaliate aggressively against affronts, and they are expected to demonstrate positive reciprocity to gifts and hospitality. In fact, Leung and Cohen (2011) found that participants from U.S. honor states who strongly endorsed honor norms were more likely than non‐honor participants to go to great lengths to return a favor. Similarly, U.S. Southerners demonstrate a stronger norm for politeness in interpersonal relationships, perhaps as means of reducing the escalation of aggression and violence that can arise from insults and affronts (Cohen et al., 1999). For example, when not insulted, Southern participants behaved more deferentially towards a stranger than did Northern participants in Cohen and his colleagues' experimental study (Cohen et al., 1996). In another study, U.S. Southerners were slower to respond with irritation to a series of annoyances from another person (but once they did respond, they were more aggressive and hostile than U.S. Northerners; Cohen et al., 1999). Similarly, Turkish participants (an honor culture) discriminated between types of offense more than did U.S. Northerners (a dignity culture): When presented with hypothetical situations that varied in the severity of the affront, Turkish participants tended to view a person who withdrew from a relatively mild affront (a rude comment) more favorably than the person who confronted, whereas the U.S. Northerners tended to evaluate withdrawal and confrontation in this situation similarly. In the absence of an insult, high‐honor participants are constructive and polite when handling a conflict situation in the workplace (Harinck et al., 2013; Shafa et al., 2015).

The role of honor values and norms in interpersonal contexts is increasingly studied in applied contexts such as organizations focusing on cooperation, collaboration, competition, and negotiation and in other social science disciplines such as economics and management (e.g., Aslani et al., 2016; Brooks et al., 2018; Gelfand et al., 2015; Günsoy et al., 2020; Ramirez‐Marin & Shafa, 2018).

## 3 HONOR IN INTRAPERSONAL PROCESSES

More recently, research has started examining how honor may shape intrapersonal processes, focusing primarily on emotional responses to honor‐relevant situations and various physical and mental health behaviors including suicide, medical help‐seeking, and risk‐taking. We review some of this research below.

### 3.1 Emotional responses to honor‐relevant situations

It has been well‐established that threats to one's honor can trigger strong emotional responses, especially among members of honor cultures (for reviews see Rodriguez Mosquera & DiBona, 2012; Uskul et al., 2019). Yet, increasing evidence has revealed that cultural differences in emotional responses vary as a function of type of threat. For example, Shafa et al. (2017) showed that Turkish participants were more likely than Dutch participants to anticipate feeling angry if someone falsely and publicly accused them of lying ‐ a threat to one's moral reputation. Dutch participants, however, were more likely to anticipate feeling angry in response to a mistake that would cause them miss out on a job promotion ‐ a threat to one's competence. More recently, Günsoy et al. (2019) showed that Turkish participants were more likely to anticipate anger and helplessness in response to reputation threats (e.g., being insulted in front of others) relative to self‐respect threats (e.g., being criticized for the things the person has done in their life), whereas the two types of threats elicited similar degrees of anger and helplessness anticipation among European Americans from Northern U.S.. Similarly, Maitner et al. (2017) showed that Arab participants whose ethnic (honor‐oriented) identity was insulted reported more anger than those whose student (non‐honor oriented) identity was insulted.

Studies that took an individual difference approach provide clear evidence for the important role played by honor in emotional responses to threatening situations. For example, Gul and Uskul (2019) asked men to imagine themselves as a primary caregiver versus breadwinner and to indicate their emotional responses to these two roles. They found that the more strongly men endorsed masculine honor beliefs, the more negative (e.g., anger, shame) and the less positive (e.g., pride, gratitude) their emotions were in response to being a caregiver (vs. breadwinner). Brand and O’Dea (2022) examined the relationship between men's endorsement of masculine honor beliefs and their reactions to their hypothetical son coming out as gay to them. The more strongly men in their study endorsed masculine honor beliefs, the more negative (e.g., embarrassment) and the less positive (e.g., pride) their self‐directed emotions were. In a similar vein, Rodriguez Mosquera et al. (2018) showed that British Muslims with strong (vs. weak) endorsement of honor were more likely to report anger in response to the cartoons that mocked prophet Muhammad, because they perceived them as more threatening to their group reputation.

### 3.2 Mental and physical health

#### 3.2.1 Honor and suicide

In cultures of honor, shame and anger following the failure to fulfill honor‐related expectations or the exposure to threats to one's personal or family reputation can turn into intrapersonal violence, especially when reputation repair through interpersonal channels is not possible. A recent analysis of case reports from the U.S. National Violent Death Reporting System showed that the suicides researchers classified as honor‐related were more likely to follow job‐related, financial, and relationship problems (Roberts et al., 2019). The analysis also showed that people who committed an honor‐related suicide were more likely to leave a note, potentially to prevent misinterpretations of their intentions, which could further damage their reputation. Honor‐related suicides are more prevalent among women (vs. men) with a Middle Eastern and North African (MENA) background (e.g., Canetto, 2015) and among White men from the U.S. honor states (vs. women and other racial groups in this region, see Crowder & Kemmelmeier, 2017; Osterman & Brown, 2011). A recent review of studies examining suicides of Turkish women in Turkey and Europe revealed that honor‐related issues, such as being accused of not being a virgin until marriage, were listed as common reasons for Turkish women's suicide attempts (e.g., van Bergen et al., 2021). In a domain that is difficult to study, these studies provide important insight into the role of honor through analysis of archival data and national statistics.

#### 3.2.2 Honor and help‐seeking for mental and physical health problems

Research has shown that people who strongly endorse honor beliefs were more likely to associate help‐seeking with weakness and a loss of reputation, and caregivers in U.S. honor (vs. other) states reported a decreased willingness to seek professional help for their children's mental health problems (Brown et al., 2014). The analysis of case reports of suicides in the U.S. also revealed that people who committed an honor‐related (vs. another type of) suicide were more likely to be depressed and yet, less likely to seek help (Roberts et al., 2019). More recently, Foster et al. (2021a) found that participants from a U.S. honor state who were more (vs. less) concerned about their reputation were more likely to have a stigma about professional help‐seeking, which was negatively associated with their intentions to seek help.

Recent research has also demonstrated that honor plays a negative role in both women's and men's physical health through reduction in willingness to seek medical help. For example, Foster and colleagues (2021b) found that women who strongly (vs. weakly) endorsed feminine honor values reported decreased intentions to schedule sexually transmitted infections screenings, because of their increased negative beliefs and emotions (e.g., shame) associated with the screening. In another study, Foster et al. (2021b) also showed that the more women valued feminine honor, the less likely they were to receive human papillomavirus (HPV) screenings, because they expected to feel shame and embarrassment if they are screened. Moreover, women with strong (vs. weak) feminine honor values were less likely to get their daughters vaccinated against HPV, because of their belief that HPV vaccination may encourage promiscuity, demonstrating that honor values can shape not only people's own health‐related choices but also those of close others'.

In relation to men's health, Foster and colleagues (2022) showed that young men with strong (vs. weak) masculine honor endorsement were more likely to have a stigma against using erectile dysfunction (ED) medication; among older men with strong masculine honor endorsement, this stigma was associated with a decreased intention to use ED medication. Moreover, ED medication prescriptions were lower in the U.S. honor states (e.g., Southern states) than in other states (e.g., Northern states). These findings suggest that the concern about losing one's masculinity can be so powerful that it may prevent some men from addressing health issues such as ED, even though addressing this would strengthen their masculine reputation in the long run.

#### 3.2.3 Honor and risky behaviors

Another line of research has focused on the association between honor values and risky health attitudes and behaviors as toughness and strength in cultures of honor can be achieved by taking risks and proving one's fearlessness. Studies have shown that risk‐taking is greater among White people from the U.S. honor (vs. other) states, manifested in greater number of accidental deaths in this region (Barnes, Brown, & Osterman, 2012; Barnes, Brown, & Tamborski, 2012) and people who strongly (vs. weakly) endorse the masculine honor ideology indicate a greater likelihood of engaging in risky behaviors (e.g., bungee jumping; Barnes, Brown, & Osterman, 2012; Barnes, Brown, & Tamborski, 2012). In another study, examining the association between several cultural constructs, including culture of honor, and engagement in risky behaviors such as not wearing a mask or not socially distancing during the pandemic, Kemmelmeier and Jami (2021) found that people from the U.S. honor states were less willing to be seen with a mask and more likely to indicate that mask‐wearing is a sign of weakness. Similarly, Schiffer et al. (2021) showed that (predominantly White) people with strong (vs. weak) masculine honor beliefs were more likely to report negative attitudes towards social distancing and more likely to be in favor of opening the economy during the pandemic.

Although these findings converge in pointing to the functional role of showing toughness and strength by taking risks and proving one's fearlessness in cultures of honor, other potential honor‐related explanations for engaging in behaviors detrimental to health remain to be studied. For example, the societal pressure of reputation management has been purported as a potential explanation for the positive association between risky alcohol and cannabis use and concerns for face and family honor among participants of MENA background in the U.S (Mechammil & Cruz, 2022).

## 4 HONOR IN INTERGROUP RELATIONS AND GROUP PROCESSES

Although research in the context of interpersonal relationships is relevant to understanding the role of honor in responses to threats, whether this research translates to the group context cannot be automatically assumed. Fortunately, researchers have recently started focusing on honor's role in intergroup relations and group processes in different world regions. In this final section, we provide a selected summary of this emerging research to underscore the added value of honor as an exploratory factor in intergroup relations and group processes.

### 4.1 Group‐based honor

One facet of honor that underscores the importance of close ingroup ties is family honor. Family honor refers to values and norms related to the protection and maintenance of the social image or the reputation of one's family and is a central part of honor in Mediterranean, Middle Eastern, and South Asian regions (e.g., Rodriguez Mosquera, 2016). In these cultures, there is a strong overlap between an individual's honor and the honor of their family; one's own actions have direct implications for their family's reputation and an individual's honor is rooted in the actions and social evaluation of their family members (Abu‐Lughod, 1999; Miller, 1993; Peristiany, 1965; Stewart, 1994).

Highlighting the centrality of family in what honor entails, research has revealed that in honor cultures (Spain, Turkey), compared to non‐honor cultures (the Netherlands, northern U.S.), honor encompasses one's family (Rodriguez Mosquera et al., 2002b), family honor is endorsed to a greater extent (Rodriguez Mosquera et al., 2002a; Shafa et al., 2015; van Osch et al., 2013), and honor‐attacking situations more frequently involve family members as targets (Uskul et al., 2012). The emphasis put on family honor in cultures of honor is also associated with important emotional, relational, and behavioral consequences, especially when family honor is at stake. For example, compared to members of a dignity culture (European‐Americans), members of cultures of honor (Pakistanis) experience more intense anger and shame and greater relationship strain when their families are insulted (Rodriguez Mosquera et al., 2014). Endorsement of honor values also predict retaliatory behavior against individuals who attack one's parents' honor in honor cultures (Turkey; Uskul et al., 2015). Moreover, individuals of Turkish origin view honor‐relevant situations as having a similar impact on one's own feelings and the feelings of family members (compared to northern Americans, who evaluate these situations as having a greater impact on one's own feelings; Uskul et al., 2012). This strong interdependence between one's personal and family's honor has been identified as forming the basis of honor‐related violence, mostly committed against female members of the family to protect and maintain the family's honor believed to be stained by real or merely alleged dishonorable conduct (Cooney, 2014; Sev'er & Yurdakul, 2001).

Going beyond the family context, recent research has also examined the role of identification with other groups (e.g., ethnic, religious, political or national groups) in how honor dynamics shape psychological processes. For example, Barnes et al. (2014) showed that the association between honor and national defensiveness (support for war on terror) was attributable in part to honor endorsers' identification with their country and their tendency to take national provocations personally. In another study, men who were strongly fused with their community (Ashokkumar & Swann, 2022) were particularly likely to endorse honor restoration as a motivation for an aggressive, punitive response to the daughter's infraction. These findings are in line with the claim that honor is most likely to be associated with identities that can provide an individual with strength and protection against rivals, which historically promoted the formation of clans or tribes (Barnes et al., 2014).

### 4.2 Honor and intergroup relations

Researchers have asked if individual or group level honor endorsement predicts aggressive responses directed to other groups (e.g., nations, terrorist groups). Barnes, Brown, and Osterman (2012); Barnes, Brown, and Tamborski (2012) showed that masculine honor ideology predicted hostile responses to a fictitious attack by foreign terrorists on the Statue of Liberty in the U.S. and support for the use of extreme counterterrorism measures such as severe interrogations, even after controlling for other relevant variables such as religious fundamentalism. In another study conducted following the terrorist attacks against the U.S. on 9/11, participants from a U.S. honor (vs. non‐honor) state wanted individuals responsible for 9/11 to be killed (Barnes, Brown, & Osterman, 2012; Barnes, Brown, & Tamborski, 2012). These findings are in line with Cohen's (1996) observation that legislators from honor states were more supportive of aggressive national security policies than their counterparts in dignity states.

Extending the above research, Saucier et al. (2018) found that masculine honor ideology predicted the perceptions of war as appropriate for the purposes of gain/revenge, protecting one's own country, protecting others, and spreading one's world‐view, as well as support for increased immigration restrictions and for detaining, torturing, and assassinating perpetrators. Masculine honor ideology also predicted people's perceptions of the world as a competitive‐jungle and their support for extreme/preemptive militarism. Similarly, Levin et al. (2015) showed that Lebanese and Syrians who valued group honor (operationalized as a general group honor concern for both one's family and nation) were more likely to perceive that the U.S. government wants to dishonor them (e.g., by disrespecting Arabs), which in turn predicted support for aggressive responses towards Americans. These findings held after controlling other relevant factors (e.g., right‐wing authoritarianism).

Political science research has also pointed to the important role played by honor in understanding intergroup relations at the international level. For example, Nawata (2020) examined whether intergroup conflict would be encountered with greater frequency in honor cultures and that this relationship would be explained by social reward for warriors. Using the Standard Cross‐Cultural Sample containing data from 186 mainly preindustrial societies from different regions of the world, Nawata found that items selected as a proxy for honor culture (e.g., revenge‐related norms) were moderately correlated with items assessing intergroup conflict (e.g., frequency of external warfare) and that this relationship was fully mediated by social rewards by warriors (e.g., prestige associated with being a soldier or warrior).

In another analysis from political science highlighting the role of reputation or status concerns in international relations, Dafoe and Caughey (2016) examined U.S. conflict behavior as a function of presidents' regional affiliation between 1816 and 2010. They tested whether leaders who were more concerned about reputation for resolve would be more likely to use military force in militarized disputes, experience longer militarized disputes, and be more likely to win and less likely to lose these disputes. Findings revealed that Southern (vs. non‐Southern) presidents were twice as likely to use force, experienced disputes that lasted on average twice as long, and three times as likely to achieve victory, which held after controlling relevant party‐related (e.g., whether the president is Democrat or Republican) and structural covariates (e.g., number of militarized interstate disputes in the last 10 years).

In a similar examination of how honor might shape political outcomes, Cohen and Leung (2012) studied the military achievement of U.S. presidents focusing on their military rank and the state (southern vs. non‐southern) from which they were nominated. They found that the only domain in which southern Presidents with military experience were rated the highest was foreign policy accomplishments. The authors interpreted this finding as reflecting the self‐help justice which develops in honor cultures that emerge in environments with weak (or absent) state‐run enforcement of rules and laws. When there are no clear authorities that nations can turn to for protection, southern presidents may be better prepared to act in international affairs as they do in internal affairs.

In addition, there is evidence demonstrating that honor can play a negative role in collective action in the context of opposition against criminal organizations (Drury & Travaglino, 2020; Travaglino et al., 2015, 2016), that political and legal structures in cultures of honor can reflect and reinforce honor‐related norms and values (e.g., more permissive gun regulations and self‐defense laws, see Cohen, 1996, Nisbett & Cohen, 1996), and that members of the military forces may be more willing to risk their lives in combat operations (Mandel & Litt, 2013). Thus, overall, research points to honor as a fruitful construct that can help understand and explain group processes and intergroup relations.

## 5 Conclusion and future directions

As this short review makes clear, the literature on honor has moved on from its initial focus on interpersonal processes, especially interpersonal aggression, to start covering a wide range of topics that provide insight into the role of honor in intrapersonal and intergroup processes whilst also incorporating a wider range of methods such as discourse analysis, diplomatic exchanges, archival documents, and memoirs of decision‐makers in addition to more conventional methods employed by social psychologists. This review also reveals that it is important to consider cultural, situational, and individual factors when drawing conclusions or forming predictions about the role of honor in intrapersonal, interpersonal, and intergroup outcomes. In line with the CuPS (Culture × Person × Situation) approach by Leung and Cohen (2011), many studies have shown that honor is likely to matter more in cultural contexts that foster the adoption of honor values, among individuals who strongly endorse these values, and in situations where honor is relevant.

Despite the growing attention that honor receives from social scientists, we believe that there is more the honor framework can do to explain various outcomes and processes including shedding important light on contemporary world events such as the role national honor may be playing in Russia's interactions with other nations (i.e., the discomfort Russia has felt due to not being recognized by the West as a great power [Smith, 2014] and as a legitimate system of values and institutions [Tsygankov, 2014]), or more generally how humiliation, status anxiety, and resentment are likely to be a significant source of conflict in honor cultures and in interaction with cultures of different value systems (e.g., Friedrichs, 2016). Future research is also needed to continue work empirically distinguishing face, dignity, and honor focusing on their similarities and differences in how they act as predictors for important intrapersonal, interpersonal, and intergroup factors and the socio‐ecological conditions under which they are likely to evolve. Finally, more research is needed on the societal and situational conditions under which honor can act as a positive or negative factor in mobilizing individuals' or groups' actions in intrapersonal, social, political, and organizational domains.

## CONFLICT OF INTEREST

All authors declare no conflict of interest.
